# Potential Applications of Fluorescence-Activated Cell Sorting (FACS) and Droplet-Based Microfluidics in Promoting the Discovery of Specific Antibodies for Characterizations of Fish Immune Cells

**DOI:** 10.3389/fimmu.2021.771231

**Published:** 2021-11-16

**Authors:** Chenjie Fei, Li Nie, Jianhua Zhang, Jiong Chen

**Affiliations:** ^1^ State Key Laboratory for Managing Biotic and Chemical Threats to the Quality and Safety of Agro-Products, Ningbo University, Ningbo, China; ^2^ Laboratory of Biochemistry and Molecular Biology, School of Marine Sciences, Ningbo University, Ningbo, China; ^3^ Key Laboratory of Applied Marine Biotechnology of Ministry of Education, Ningbo University, Ningbo, China

**Keywords:** teleost fish, immune cells, monoclonal antibodies, fluorescence-activated cell sorting, droplet-based microfluidics

## Abstract

Akin to their mammalian counterparts, teleost fish possess a complex assortment of highly specialized immune cells that are capable of unleashing potent innate immune responses to eradicate or mitigate incoming pathogens, and also differentiate into memory lymphocytes to provide long-term protection. Investigations into specific roles and functions of fish immune cells depend on the precise separation of each cell type. Commonly used techniques, for example, density gradient centrifugation, rely on immune cells to have differing sizes or densities and thus fail to separate between similar cell types (e.g. T and B lymphocytes). Furthermore, a continuously growing database of teleost genomic information has revealed an inventory of cellular markers, indicating the possible presence of immune cell subsets in teleost fish. This further complicates the interpretation of results if subsets of immune cells are not properly separated. Consequently, monoclonal antibodies (mAbs) against specific cellular markers are required to precisely identify and separate novel subsets of immune cells in fish. In the field of fish immunology, mAbs are largely generated using the hybridoma technology, resulting in the development of mAbs against specific cellular markers in different fish species. Nevertheless, this technology suffers from being labour-intensive, time-consuming and most importantly, the inevitable loss of diversities of antibodies during the fusion of antibody-expressing B lymphocytes and myeloma cells. In light of this, the focus of this review is to discuss the potential applications of fluorescence-activated cell sorting and droplet-based microfluidics, two emerging technologies capable of screening and identifying antigen-specific B lymphocytes in a high-throughput manner, in promoting the development of valuable reagents for fish immunology studies. Our main goal is to encourage the incorporation of alternative technologies into the field of fish immunology to promote the production of specific antibodies in a high-throughput and cost-effective way, which could better allow for the precise separation of fish immune cells and also facilitate the identification of novel immune cell subsets in teleost fish.

## Introduction

Teleost fishes represent a large and diverse group of vertebrates thriving in an extremely dynamic range of aquatic ecosystems that are populated with numerous parasitic, bacterial, and viral pathogens. Conceivably, a robust immune system has evolved to deal with these continuous assaults. A critical component in teleost immunity is specialized immune cells and it has been reported that immune cells isolated from fish are capable of preforming a range of effector responses that are indistinguishable from their mammalian counterparts (1–4). Investigations into functions and roles of teleost immune cells have revealed interesting discoveries, including the identification of a novel antibody isotype (i.e. IgT) that is unique to fish, and also B cells exhibiting phagocytic capabilities that later on are found to be evolutionarily conserved features (2, 5). These observations highlight the importance of using teleost fish as model organisms to study conserved and also divergent aspects of immunity across evolution.

The key first step in investigating cellular immunity is the precise separation of immune cells of interest. In the field of teleost immunology, this is commonly achieved *via* density gradient centrifugation. Generally, this technique involves layering a mixture of cells on gradient density medium (e.g. Ficoll^®^-Paque and Percoll) and after centrifugation, cells of higher buoyant density (i.e. erythrocytes and granulocytes) pellet to the bottom whereas mononuclear cells (i.e. macrophages/monocytes and lymphocytes) settle in an interphase layer (6–11). Afterwards, an added separation process can be applied to further purify cells of interest. For example, macrophages/monocytes can be further purified to eliminate lymphocytes based on different adherent properties (12–15). Of note, cell types of similar densities, such as T and B lymphocytes, are unlikely to be separated due to the nature of this technique. Furthermore, a growing number of reports demonstrated that subsets of immune cells with differential functions in immunity are also present in teleost fish (16–19). For example, similar to their mammalian counterparts, CD4 and CD8 molecules have been identified in a range of teleost species, indicating the presence of helper and cytotoxic T lymphocytes (16, 17). However, it is important to not assume that teleost immune cells will express the same surface markers as mammals do. Teleost immune cell subsets could also be determined by the expression of membrane-bound molecules that are unique to fish (e.g. novel immune type receptors) or evolutionally conserved (e.g. channel catfish leukocyte immune-type receptors) or secretions of cytokines (20–23). This further illustrates the complexity of cellular components in teleost immunity and a detailed characterization of immune cell subsets is required to further dissect specific roles of each cell type/subset, which can only be achieved when they are properly separated. Overall, density gradient centrifugation is a useful technique to separate cells exhibiting varying densities, but it fails to resolve cell types of similar physical characteristics.

To characterize teleost immune cells with better resolution, alternative methods like antibody-based separation techniques are developed to isolate cells of interest. These techniques take advantage of monoclonal antibodies (mAbs) that are capable of recognizing epitopes with high specificity and thus, allowing to label cells of interest for precise separation. Indeed, mAbs targeting known immune cell markers in model organisms (e.g. mammals) have been successfully developed through various technologies. In teleost research, however, these valuable reagents are limited to a few species and largely generated through traditional hybridoma-based technology. A detailed summary of available reagents targeting well-characterized surface markers and immune cell types in teleost fish is presented in **Table 1**. The scope of this article is to review currently available technologies for the production of mAbs. Specifically, the hybridoma technology, fluorescence-activation cell sorting (FACS) and droplet-based microfluidics technologies with an emphasis on the latter two. Other methods, like phage display technology is not included in this review due to inherently different antibody-producing mechanisms, and readers interested in this specific technology are referred to excellent reviews elsewhere (99, 100). Our main goal is to provide alternative options for the development of mAbs in this field, which will be the basis of identifying and separating immune cell types and characterizing respective roles to further our understanding of teleost cellular immunity.

**Table 1:** An overview of available reagents targeting surface markers/immune cells in teleost fish.

Specificity					Fish species	Available reagents	Technologies	REF
					Asian seabass (*Lates calcarifer*)	mAbs*	Hybridomas	(24)
					Atlantic cod (*Gadus morhua L.*)	mAbs	Hybridomas	(25)
					Atlantic salmon (*Salmo salar*)	mAbs	Hybridomas	(26)
					Bighead catfish (*Clarias macrocephalus*)	mAbs	Hybridomas	(27)
					Black rockfish (*Sebastes schlegeli Higendorf*)	mAbs	Hybridomas	(28)
					Brown trout (*Salmo trutta*)	mAbs*	Hybridomas	(29)
					Channel catfish (*Ictalurus punctatus*)	mAbs	Hybridomas	(30)
					Common carp (*Cyprinus carpio L.*)	mAbs	Hybridomas	(31)
					European eel (*Anguilla anguilla L.*)	mAbs	Hybridomas	(32)
					European sea bass (*Dicentrarchus labrax L.*)	mAbs	Hybridomas	(33)
					Flounder (*Paralichthys olivaceus*)	mAbs	Hybridomas	(34, 35)
					Gibel carp (*Carassius auratus gibelio*)	mAbs	Hybridomas	(36)
					Gilthead seabream (*Sparus aurata*)	mAbs	Hybridomas	(37)
					Half-smooth tongue sole (*Cynoglossus semilaevis*)	mAbs	Hybridomas	(38)
					Large yellow croaker (*Larimichthys crocea*)	mAbs	Hybridomas	(39, 40)
Immunoglobulins					Mrigal carp (*Cirrhinus mrigala*)	mAbs	Hybridomas	(41)
					Muskellunge (*Esox masquinongy*)	mAbs	Hybridomas	(42)
					Nile tilapia (*Oreochromis niloticus*)	mAbs	Hybridomas	(24, 43, 44)
					Pacific herring (*Clupea pallasii*)	mAbs	Hybridomas	(45)
					Rainbow trout (*Oncorhynchus mykiss*)	mAbs	Hybridomas	(46, 47)
					Red drum (*Sciaenops ocellatus*)	mAbs	Hybridomas	(48)
					Rohu (*Labeo rohita*)	mAbs	Hybridomas	(49)
					Sea bass (*Lateolabrax japonicus*)	mAbs	Hybridomas	(50)
					Sevenband grouper (*Epinephelus septemfasciatus*)	mAbs	Hybridomas	(51)
					Smallmouth bass (*Micropterus dolomieu*)	mAbs	Hybridomas	(52)
					Snakehead (*Channa striata*)	mAbs	Hybridomas	(53)
					Snapper (*Pagrus auratus*)	mAbs	Hybridomas	(54)
					Torafugu (*Takifugu rubripes*)	mAbs	Hybridomas	(55)
					Turbot (*Scophthalmus maximus*)	mAbs	Hybridomas	(56)
					Walking catfish *(Clarias batrachus)*	mAbs	Hybridomas	(57)
					White sturgeon (Acipenser transmontanus)	mAbs	Hybridomas	(58)
					Flounder (Paralichthys olivaceus)	mAbs	Hybridomas	(59)
CD3/TCR					Rainbow trout (*Oncorhynchus mykiss*)	mAbs	Hybridomas	(46, 60)
					Grass carp (*Ctenopharyngodon idella*)	mAbs	Hybridomas	(61)
					Flounder (*Paralichthys olivaceus*)	mAbs	Hybridomas	(62, 63)
					Ginbuna crucian carp (*Carassius auratus langsdorfii*)	mAbs	Hybridomas	(17)
CD4									
					Rainbow trout (*Oncorhynchus mykiss*)	mAbs	Hybridomas	(64)
					Zebrafish (*Danio rerio*)	Transgenic fish line	Genome editing	(65)
					Atlantic salmon (*Salmo salar*)	mAbs	Hybridomas	(66)
CD8					Ginbuna crucian carp (*Carassius auratus langsdorfii*)	mAbs	Hybridomas	(67)
					Rainbow trout (*Oncorhynchus mykiss*)	mAbs	Hybridomas	(68)
					Cempedic fish (*Osteochilus spilurus*)	mAbs	Hybridomas	(69)
					Common carp (*Cyprinus carpio L*.)	mAbs	Hybridomas	(70–73)
Macrohpages/monocytes					Flounder (*Paralichthys olivaceus*)	mAbs	Hybridomas	(74)
					Rainbow trout (*Oncorhynchus mykiss*)	mAbs	Hybridomas	(75, 76)
					Yellowtail (*Seriola quinqueradiata*)	mAbs	Hybridomas	(77)
					Zebrafish (*Danio rerio*)	Transgenic fish line	Genome editing	(78–80)
					Atlantic salmon (*Salmo salar*)	mAbs	Hybridomas	(81)
					Ayu (*Plecoglossus altivelis*)	mAbs	Hybridomas	(82)
					Channel catfish (*Ictalurus punctatus*)	mAbs	Hybridomas	(83)
					Common carp (*Cyprinus carpio L*.)	mAbs	Hybridomas	(73, 84)
					European sea bass (*Dicentrarchus labrax L*.)	mAbs	Hybridomas	(85)
Lymphocytes					Indian Carp (*Catla catla*)	mAbs	Hybridomas	(86)
					Rainbow trout (*Oncorhynchus mykiss*)	mAbs	Hybridomas	(87)
					Snapper (*Pagrus auratus*)	mAbs	Hybridomas	(88)
					Yellowtail (*Seriola quinqueradiata*)	mAbs	Hybridomas	(77)
					Zebrafish (*Danio rerio*)	mAbs*	Hybridomas	(89)
					Zebrafish (*Danio rerio*)	Transgenic fish line	Genome editing	(90)
					Atlantic salmon (*Salmo salar*)	mAbs	Hybridomas	(81)
					Ayu (*Plecoglossus altivelis*)	mAbs	Hybridomas	(82)
					Common carp (*Cyprinus carpio L*.)	mAbs	Hybridomas	(71)
					Channel catfish (*Ictalurus punctatus*)	mAbs	Hybridomas	(83)
Neutrophils					Flounder (*Paralichthys olivaceus*)	mAbs	Hybridomas	(74)
					Gilthead seabream (*Sparus aurata L*.)	mAbs	Hybridomas	(91)
					Rainbow trout (*Oncorhynchus mykiss*)	mAbs	Hybridomas	(76)
					Yellowtail (*Seriola quinqueradiata*)	mAbs	Hybridomas	(77)
					Zebrafish (*Danio rerio*)	Transgenic fish line	Genome editing	(92)
					Ayu (*Plecoglossus altivelis*)	mAbs	Hybridomas	(82)
					Blue catfish (*Ictalurus furcatus*)	mAbs	Hybridomas	(93)
					Common carp (*Cyprinus carpio L.*)	mAbs	Hybridomas	(94)
Thrombocytes					Channel catfish (*Ictalurus punctatus*)	mAbs	Hybridomas	(93)
					Flounder (*Paralichthys olivaceus*)	mAbs	Hybridomas	(74)
					Rainbow trout (*Oncorhynchus mykiss*)	mAbs	Hybridomas	(95, 96)
					zebrafish (*Danio rerio*)	Transgenic fish line	Genome editing	(97, 98)

A literature search of available reagents, specifically mAbs and transgenic fish lines, that target specific surface markers and immune cell types in teleost fish was performed. Reagents like polyclonal antibodies are not included in this table.

*mAbs that were not originally developed against listed species but were later shown cross-reactivity were asterisked.

## Hybridoma Technology

Antibodies, also known as immunoglobulins, are glycoproteins exclusively produced by B cells and come in two forms. Specifically, membrane-bound antibodies (e.g. B cell receptors) that normally expressed on the surface of naïve B cells to recognize foreign antigens, and soluble antibodies, for example, immunoglobulin G (IgG) that secreted by mature B cells after undergoing somatic hypermutations and class switching. Each species of antibodies specifically recognize epitopes of the same structure and this specificity of antibodies makes them valuable tools in therapeutics, diagnosis and research (101, 102). Currently, most of available antibodies on the market is IgG isotype since it exhibits the highest binding affinity to cognate antigens and thus, antibodies mentioned below are all referring to IgG isotype unless stated otherwise. It has been a daunting challenge to culture mature B lymphocytes *in vitro* for the continuous production of antibodies. Consequently, numerous resources and efforts have been invested to establish and optimize platforms for screening and culturing B lymphocytes secreting antibodies with desired specificity and affinity. Hybridoma technology represents the first platform for the continuous production of mAbs with known specificity and affinity since the invention in 1975 (103). This technology involves the fusion of splenic B cells isolated from immunized animals with an immortal B cell line (i.e. myeloma cells). These hybrid cells, called hybridomas, are immortal as myeloma cells while still maintain the ability of immunized splenic B cells to produce antibodies of interest. These hybridomas are further cloned and secreted mAbs are characterized in binding assays (e.g. ELISA) to select for hybridoma cell lines producing desired mAbs. Since each hybridoma cell line normally secrets a single species of antibodies, selected hybridoma cell lines could then serve as a continuous source for the production of mAbs with known specificity and affinity.

Currently, hybridoma technology remains the most popular method for generating mAbs and in the field of teleost immunology, majority of reported mAbs targeting surface markers and immune cell lineages were generated using this technology (**Table 1**). That being said, it is important to note that there are several inherent limitations pertaining to this technology. As mentioned above, generation of hybridomas requires the fusion of antigen-specific B cells and myeloma cells, which is commonly achieved using chemical reagents, like polyethylene glycol. This chemical reagent dehydrates lipid head groups on the cell membrane, resulting in an asymmetry of the membrane bilayer that favours the fusion of two adjacent cells. However, this fusion process is non-selective that often leads to the fusion of the same kind of cells and thus, result in the loss of antigen-specific B cells. Although variants of hybridoma technology have been developed to improve fusion efficiency, for example the B cell targeting method, repertoire of mAbs obtained using this technology is still biased since only B cells survive the fusion process could be selected (104). This largely reduces antibody diversity and consequently, selected mAbs are less likely the one with the optimal specificity and affinity. Furthermore, mAbs secreted by hybridomas are not always monospecific as assumed and gene expression of additional heavy and/or light chains was found within individual hybridomas (105). Collectively, these inherent limitations prevent the application of hybridoma technology in certain settings and with the advent of novel technologies, new platforms have emerged as alternative options for the production of mAbs, which are discussed below.

## FACS and Droplet-BASED Microfluidics as Alternative Technologies for the Rapid Production of mAbs

**Figure 1:**
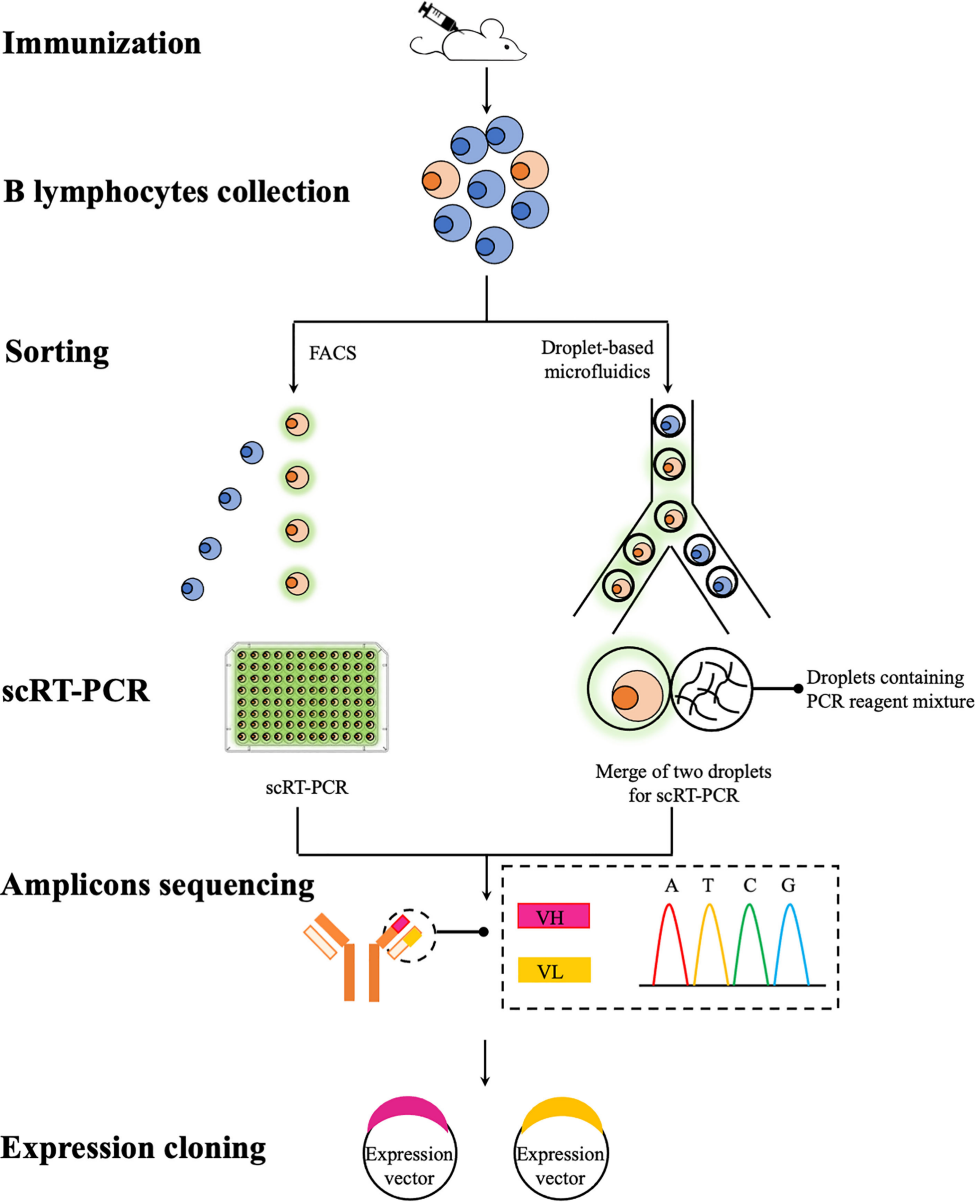
A schematic overview of recombinant mAbs production using FACS and droplet-based microfluidics. B lymphocytes (e.g. splenocytes and plasmablasts) are collected from immunized mice and then individually sorted *via* FACS or droplet-based microfluidics for antigen-specific lymphocytes. Paired VH and VL sequences are then amplified using scRT-PCR for subsequent sequencing (e.g. traditional Sanger sequencing and next-generation sequencing if bar-coded primers used during the scRT-PCR step) and subcloning. Expression vectors containing VH and VL sequences can be co-transfected into a range of expression systems for the production of recombinant mAbs.

A common theme in any mAbs discovery campaigns is to identify and isolate single B cells expressing and secreting mAbs with desired specificity and affinity. This posts a daunting task as the pool of antigen-specific B cells is highly heterogenous that each B cells normally secretes a single species of antibodies with unique properties (106). Although it is practically difficult, if not impossible, to screen all available B cells, various high-throughput technologies have been developed that allow a greater number of B cells to be screened, thus increasing the chance of finding optimal mAbs. FACS and droplet-based microfluidics, represent two high-throughput technologies for the *in vitro* screen of B cells of interest (**Figure 1**). Unlike traditional hybridoma technology that requires the immortalization of antibody-expressing B cells before any type of analysis, these two technologies offer unique opportunities to screen a repertoire of primary B cells that can be individually sorted in a high-throughput fashion. Sorted B cells are further analyzed, normally to obtain sequences of immunoglobulins heavy and light chain variable regions (VH and VL) since the binding specificity and affinity of immunoglobulins are largely determined by these two regions (106). These obtained sequences could then be subcloned and expressed for the production of recombinant mAbs. Using these two technologies coupled with single cell expression cloning, numerous success in the rapid identification and generation of rare mAbs species against a range of infectious agents has been reported in mammalian research (107–109). To date, very few teleost studies exploit these technologies for the generation of mAbs and therefore in this section, we will review these two technologies and also their potential applications in generating specific mAbs for teleost immunology studies.

## FACS

Modern cell sorter (a type of flow cytometer capable of analyzing and also sorting cells of interest) is a multi-parameter instrument that allows the simultaneous measurement of optical and fluorescence characteristics of each single cell. Parameters, such as size, granularity and fluorescence features derived from fluorescent dyes bound to cells, are commonly used to differentiate and sort cells of interest. To date, a sophisticated cell sorter is capable of sorting up to thousands of cells per second while maintain reasonable viability. This makes it a valuable tool to efficiently discriminate B cells of interest from a large pool of highly heterogenous cells for further analysis at a single cell level.

Generally speaking, there are two strategies identifying B cells of interest using FACS for subsequent recombinant mAb production. One is antigen baiting that involves the use of fluorescently labelled antigens as a bait to sort antigen-specific B cells. This strategy is amenable to subsets of B cells expressing surface-bound antibodies (e.g. memory B cells) and often obtained recombinant mAbs exhibit desired specificity (110, 111). An alternative strategy relies on the fluorescent staining of defined surface markers, allowing to identify and isolate a broader spectrum of B cell subsets. This is particularly useful for identifying short lived plasmablasts that represent a transient population of antigen-specific B cells, normally observed 5~8 days after immunization (112–114). Of note, plasmablasts are unlikely to be identified using antigen baiting due to the low expression levels of surface-bound antibodies. Overall, these two FACS-based strategies allow an efficient and selective isolation of individual antigen-specific B cells for further analysis.

After FACS of antigen-specific B cells of interest, the next step is to obtain sequence information of paired VH and VL genes of each cell. This is often achieved through variants of polymerase chain reactions (PCR) coupled with different sequencing platforms. Single cell reverse transcription PCR (scRT-PCR) represents a major advance in single cell technology and allows to amplify genes of interest from a single cell (110). Sorted antigen-specific B cells are lysed in microtiter plates and after reverse transcription, cDNA templates of single cells are collected for amplification of VH and VL genes followed by Sanger sequencing. This combination of technologies was first employed to study autoantibody production during B cell development (115). Since then it has been adapted to antibody discovery campaigns and has led to the successful identification of neutralizing mAbs against a range of viral pathogens, including HIV and influenza virus (107, 108). However, this system is inherently low throughput since each sorted cell has to be processed and sequenced individually and practically, a few hundred VH and VL pairs at most could be identified using this system (116–118). This number is dwarfed by the enormous size of antibody repertoire that potentially, >10^15^ possible antibodies recognizing unique binding sites could be generated in mouse and human (119). More recently, a smart design of two-dimensional primer matrix largely improves the throughput of scRT-PCR; amplicons (e.g. paired VH and VL genes) from the same cell are bar-coded and thus, all amplicons could be pooled and analyzed using next generation sequencing (NGS) technology. This increased the number of analyzed B cells up to ~50,000 per experiment (120). Alternatively, the idea of linking VH and VL segments from single antigen-specific B cells was revisited and has been achieved *via* emulsion over-extension linkage RT-PCR. In this method, antigen-specific B cells are deposited by gravity into microwell arrays, in which individual cells are lysed and mRNAs are captured by poly(dT) beads in microwells. mRNAs-poly(dT) beads complexes are further washed and emulsified with primers, reverse transcriptase and polymerase to perform reverse transcription followed by linkage RT-PCR and Illumina sequencing (121). This method allows to screen >50,000 antigen-specific B cells per experiment with the potential to further scale up.

After obtaining sequencing information, paired VH and VL sequences originating from single antigen-specific B cells are then could be subcloned and expressed to produce recombinant mAbs. Numerous expression systems using eukaryotic and prokaryotic hosts have been developed (122). Each system is balanced by advantages and drawbacks and the choice of particular systems largely relies on applications of recombinant mAbs, which is detailed reviewed elsewhere (106). Overall, This FACS-based platform coupled with single cell sequencing and expression cloning has facilitated an efficient identification of antigen-specific B cells that leads to a rapid generation of recombinant mAbs. That being said, selecting paired VH and VL candidates for subsequent expression and validation of recombinant mAbs still presents a tedious task; furthermore, this platform primarily screens for antigen-specific B cells based on antibody binding specificity while other information regarding to antibodies, such as binding affinity and functional characteristics (e.g. neutralizing capacity), is not available until recombinant mAbs are generated. More recently, droplet-based microfluidics system has emerged as a cutting-edge platform and gradually established as a valuable tool for a range of applications, including single cell analysis with improved resolution, and will be discussed below.

## Droplet-Based Microfluidics

**Figure 2:**
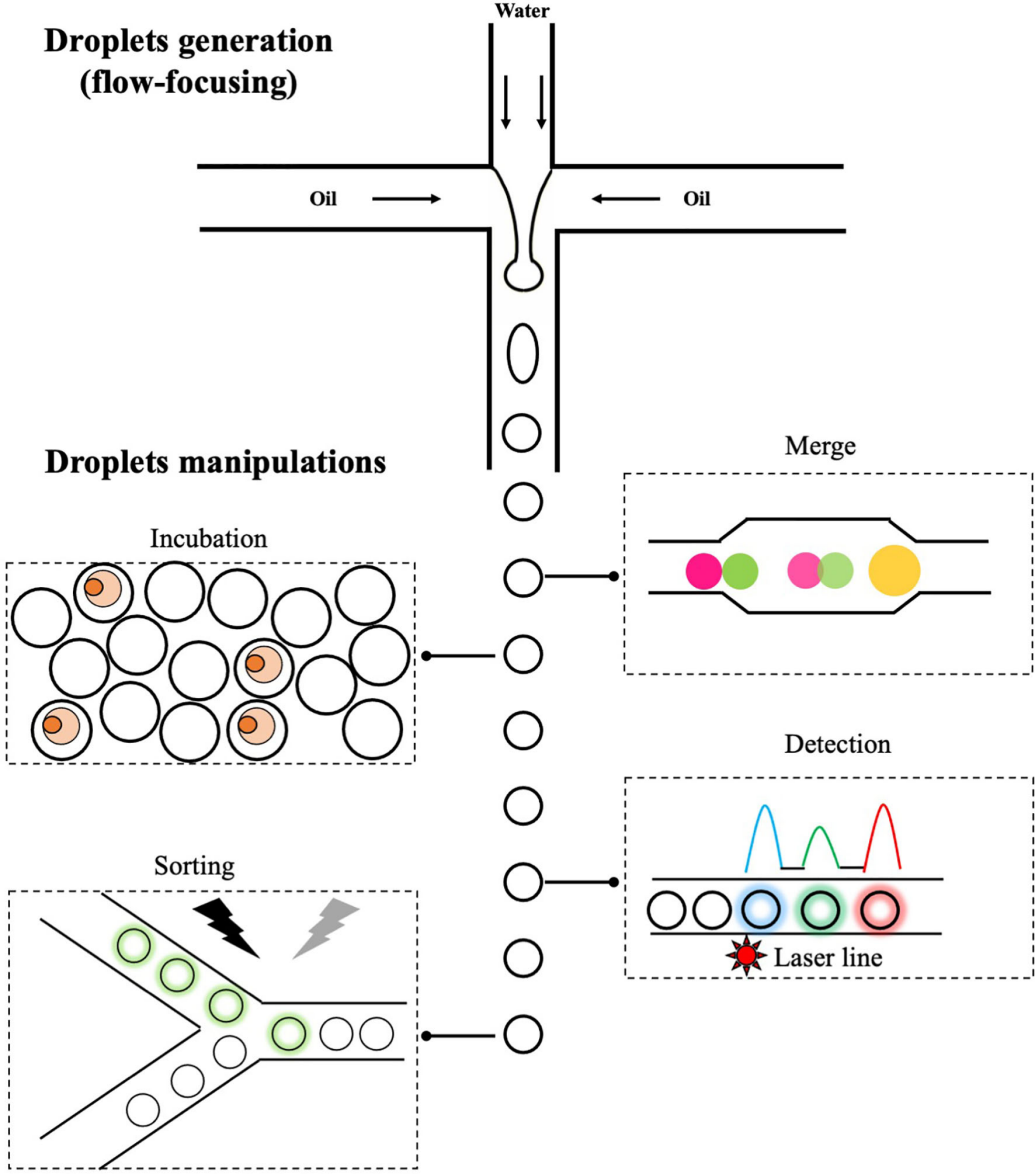
The generation and manipulations of droplets. One of popular methods to generate droplets in microfluidics is flow-focusing, in which the injected aqueous stream (water phase) is sheared by perpendicular oil streams pumped from two side channels. Once fluids meet, water in oil droplets are formed and those droplets can be further manipulated: merge of two droplets with different contents, on/off-chip incubation of droplets to allow reactions to occur, detection of fluorescent signals and sorting of droplets of interest *via* electric field.

Microfluidics is a technology that manipulates the flow of fluids through microchannels. At microscales, fluids behave very differently and give rise to unique features that are useful for a range of applications (123, 124). One branch of microfluidics is droplet-based microfluidics, which involves precise controls of two immiscible liquids (e.g. water and oil) through microchannels and leads to the formation of highly monodisperse aqueous droplets flowing in the carrier oil (**Figure 2**). These droplets, normally ranging from pico- to nanoliters, are functionally equivalent to individual wells in microtiter plates but with significantly reduced size, and biological entities (e.g. cells) encapsulated in each droplet are chemically and physically separated. Once formed, droplets could be further manipulated in a variety of ways (**Figure 2**). For example, addition of new reagents through droplets merge (125); static on/off-chip incubation of droplets (126, 127); detection of fluorescent signals in droplets and selective sorting of droplets of interest using electric field (128). The small volume of droplets combined with various ways of manipulation offers unique opportunities to study single cell biology in an unprecedent way. For example, secreted proteins are co-encapsulated with single cells inside droplets and quickly reach detectable concentrations due to the small droplet volume, resulting in a rapid detection of molecules/cells of interest. This is particularly useful in isolating plasmablasts (a subset of antibody-secreting B cells with low expression level of surface-bound antibodies) and overcomes limitations of aforementioned antigen baiting strategy. In addition, encapsulated cells could be lysed and intracellular molecules assayed within droplets; one example is the reverse transcription and amplification of genetic materials. This enables a more comprehensive analysis of single cells at biochemical and genetical levels. In antibody discovery campaigns, these features offer a unique opportunity to link genotype (e.g. sequencing information of VH and VL regions) with phenotype (e.g. properties of antibodies, such as specificity and affinity) in a one-stop manner. Indeed, various droplet-based microfluidics platforms have been developed and allows to characterize B cells of interest with improved resolution.

**Figure 3:**
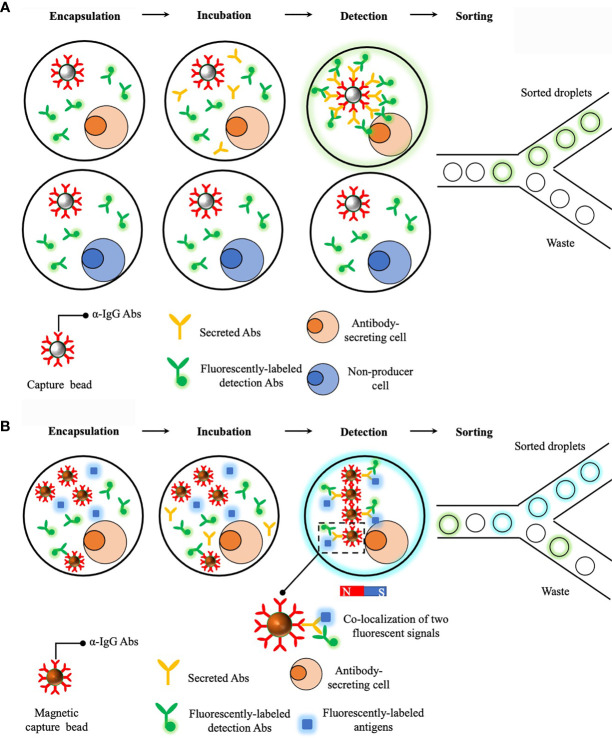
Droplet-based microfluidics platforms for single cell antibodies secretion measurements. **(A)** Sorting of antibody-secreting cells. Secreted antibodies were captured on the bead together with fluorescently-labeled detection antibodies. This leads to a localization of fluorescent signals on the bead and fluorescent droplets can then be sorted. **(B)** Dropmap platform for studying antibody secretion and affinity to cognate antigens. Compared to the previous platform, a major improvement of Dropmap is the encapsulation of magnetic beads and fluorescently-labeled antigens within each droplets. After antibody secretion, a magnetic field is applied to induce the formation of elongated and easily observable bead aggregates line, termed beadline. This helps amplify fluorescent signals and therefore, increase the sensitivity. Normally there are three scenarios after incubation, the first one is no fluorescent signal is observed and this indicates the cell is not able to secret antibodies; the second scenario is that only fluorescent signals from detection antibodies is localized on magnetic beads and this indicates the cell is able to secret antibodies but with poor specificity and affinity to cognate antigens; lastly, co-localization of two fluorescent signals is observed and this indicates cell is able to secret antibodies with desired specificity and affinity to cognate antigens.

A prototype of droplet-based microfluidics platform that enables a rapid detection of secreted antibodies was developed in 2013 and demonstrated as a powerful tool to sort antibody-secreting cells in a high-throughput way (129). Briefly, single cells are encapsulated with a capture bead (i.e. beads are opsonized with anti-IgG antibodies) and fluorescent probes (i.e. detection antibodies specifically targeting antigen-binding fragment (Fab) regions of IgGs). This is essentially a sandwich enzyme linked immunosorbent assay (ELISA) detecting secreted proteins from single cells and after 15min incubation, secreted antibodies are captured on the bead, together with localized fluorescent probes, and result in a clearly distinguishable increase in fluorescent intensity on beads and thus, droplets containing antibody-secreting cells can be sorted (**Figure 3A**). This represents a major advance in identifying antibody-secreting cells within a short time frame and could be potentially incorporated with other technologies to retrieve the genotype. However, this prototype fails to obtain useful information regarding to secreted antibodies, such as binding specificity and affinity, and therefore limit its applications in screening antigen-specific B cells.

An updated version of aforementioned prototype, termed DropMap, was further developed with features of determining antibody affinity to specific antigens (130). A major improvement of this updated platform is the co-encapsulation of single cells, antigens and detection antibodies that are differentially labeled, together with multiple magnetic capture beads (**Figure 3B**). Similar to the previous prototype, secreted antibodies are captured on beads, together with detection antibodies and potentially, antigens. Then a magnetic field is applied to induce the formation of an elongated and easily observable bead aggregates line, termed beadline, within each droplet. Consequently, binding specificity and affinity of secreted antibodies are determined by analysis of co-localization of fluorescent signals on the beadline and also the measurement of fluorescent intensity of antigens localized to the beadline, respectively. This DropMap platform allows a simultaneous measurement of antibody secretion rate, specificity and affinity, and this likely potentiates antibody screening process. Most recently, a novel platform, termed Celli*GO*, that integrates DropMap with NGS technology was established (131). In this platform, droplets containing single cells of interest (i.e. cells secreting antibodies with desired specificity and affinity) are sorted and merged with droplets containing PCR reagents. Paired VH and VL regions derived from single cells are amplified using bar-coded primers within droplets before NGS analysis. This platform seamlessly integrates multiple steps, ranging from sorting single cells, characterizing properties of secreted antibodies and obtaining sequencing information that are normally performed separately, into one device. Combined with NGS, this Celli*GO* platform represents a great advance in coupling phenotype with genotype of antigen-secreting cells and exhibits great potentials in discovering rare antigen-specific antibodies against difficult targets.

Although successful development of desired mAbs has been seen using droplet-based microfluidics, its applications have been largely limited to translational research so far using mammalian models due to the cost of this platform (e.g. expensive droplet-based microfluidics systems combined with the cost on regular maintenance and costly consumables). One example is to identify neutralizing mAbs against infectious agents, which have great therapeutic potentials (132, 133). Currently, it is economically impractical to apply this technology to generate mAbs reagents that are sorely for basic immunology research in non-model species. However, this still represents an alternative option and can be taken into account if specific mAbs reagents against difficulty targets are urgently required to address interesting hypotheses.

## Potential Applications of FACS and Droplet-based Microfluidics Technologies for Teleost Immunology Research

Comparative immunology studies using non-model organisms like teleost fish have been expanding our understanding of immune systems and uncover interesting discoveries that are unique to fish or mechanistically conserved across evolution (2, 5). Most of these findings in teleost immunology studies are partially attributing to the availability of specific mAbs that allow the identification and isolation of different immune cell types. One example is the discovery of phagocytic B cells in rainbow trout, which would be impossible if no mAbs against rainbow trout IgM was available (2). In mammalian research, panels of mAbs targeting most of known immune-related molecules (e.g. surface markers) are commercially available. This enables precise isolations of different immune cell types, which is the key first step in further understanding specific roles of each cell type under different contexts and also how immune system operates in general. In comparison, these valuable reagents are mostly produced in-house and only limited to a few teleost species. Although teleost fish possess a variety of immune cells that are actively involved in innate and adaptive immunity, detailed functional characterizations of different immune cell types in ongoing immune responses is largely unavailable due to the lack of mAbs of high quality. To date, most of available mAbs in teleost immunology studies were generated using the hybridoma technology that requires lengthy immunization processes and labour-intensive cell fusion, cloning and screening procedures, which to some extent, discouraging investigators from generating these reagents. Currently, the advent in FACS and microfluidics technologies provide alternative options for a rapid identification of antigen-specific cells and thus, lead to the generation of mAbs in a more efficient way.

One of challenges associated with teleost fish for immunology studies is the presence of multiple copies of immune-related genes due to a teleost-specific whole genome duplication (WGD) events followed by a lineage-specific WGDs in several fish groups (e.g. salmonids and cyprinids) (134). This leads to an extensive expansion of genes encoding for immune receptors, cytokines and other components involved in immunity in teleost fish (135–144). For example, several isoforms of CD4 molecules differing slightly in extracellular domain structure have been identified in a range of fish species as opposed to only one copy of CD4 gene in human (145–148). This adds an additional layer of complexity in terms of reagents development, which requires mAbs with extreme specificity to differentiate isoforms that could potentially be identifiers of novel immune cell subsets. In this regard, FACS combined with scRT-PCR can potentially serve as a high-throughput platform to sort antigen-specific B cells via antigen-baiting for subsequent mAbs production. In addition to antigens of interest, antigens derived from other duplicated genes can also be differentially labeled with fluorochromes and serve as controls to exclude B cells exhibiting cross-reactivity during the sorting process, which will largely increase the chance of obtaining individual B cells with high specificity for subsequent scRT-PCR and expression cloning. In comparison to traditional hybridoma technology, this platform is more selective and avoids unnecessary waste (e.g. reagents and time) on non-producer/specific hybridomas and thus, improve the efficiency of developing mAb targeting closely-related isoforms in teleost fish. Furthermore, cytokine genes are also expanded in teleost fish and in the context of immunology, multiple copies of pro-/anti-inflammatory cytokine genes are present and responsible for shaping ongoing immune responses (149, 150). To date, teleost fish cytokines are mostly examined at transcript level and data regarding expression of cytokine molecules under resting state/upon stimulations is scarce due to the lack of mAb reagents (151). Besides, expression levels of cytokine molecules are relatively low under physiological conditions (151). These scenarios combined make it fairly difficult to generate mAbs reagents that are satisfying the requirement (mAbs exhibiting extremely high specificity and affinity) to allow sensitive and accurate detections of teleost cytokines. In this regard, droplet-based microfluidics combined with NGS is a better alternative for identifying antigen-specific B cells for subsequent sequencing and expression cloning. Properties of secreted antibodies derived from individual B cells can be parallelly analysed within droplets to retrieve optimal antigen-specific B cells. This platform provides better resolutions to allow a stringent selection of B cells based on both specificity and affinity of secreted antibodies and will largely reduce efforts and time spent in screening and validating generated mAbs afterwards and thus, increase the likelihood of obtaining desired mAbs against these difficulty targets.

Overall, the choice of particular technologies for mAb development is dependent on multiple factors, such as the applications/requirements of mAbs and availability of specialized equipment. There is no doubt that integration of novel technologies into our field will aid in the development of mAbs in a more efficient way, which is the basis of interesting discoveries in teleost immunology studies.

## Conclusions

Teleost fishes represent the first species with an adaptive immunity and have been valuable model species in comparative immunology for understanding the evolution of immune systems. Knowledge obtained from teleost immunology studies have led to discoveries that were previously not aware of through studies of mammalian models (e.g. human and mouse). Despite their critical places in the evolutionary tree, teleost immunology studies are still at an early stage and in-depth characterizations of teleost immune cell types and their cytokine secretion profiles remain to be established primarily due to the lack of mAbs reagents of high quality, which largely prevents a further understanding of how immune systems operate in fish. Since the development of mAbs in this field largely relies on hybridoma technology, incorporation of available technologies, such as FACS and droplet-based microfluidics, into this process should be taken into account as this would largely increase the efficiency of mAbs production and eventually, lead to investigations on a broader spectrum of teleost species which may reveal novel immune cell types/subsets with distinct functions.

## Author Contributions

CF, LN, and JC wrote and edited the manuscript. JZ provided the data and edited the manuscript. All authors contributed to the article and approved the submitted version.

## Funding

This work was supported by following grants: a Natural Science Foundation of Zhejiang Province (LZ18C190001); a Program for the Natural Science Foundation of China (31972821); a Natural Science Foundation of Ningbo (202003N4011; 20211JCGY020201) and a research fund of Ningbo University (422109623).

## Conflict of Interest

The authors declare that the research was conducted in the absence of any commercial or financial relationships that could be construed as a potential conflict of interest.

## Publisher’s Note

All claims expressed in this article are solely those of the authors and do not necessarily represent those of their affiliated organizations, or those of the publisher, the editors and the reviewers. Any product that may be evaluated in this article, or claim that may be made by its manufacturer, is not guaranteed or endorsed by the publisher.
